# Toward the Design of Sensing-Based Medication Adherence Aids That Support Individualized Activities of Daily Living: Survey and Interviews With Patients and Providers

**DOI:** 10.2196/40173

**Published:** 2023-07-04

**Authors:** Jacob T Biehl, Ravi Patel, Adam J Lee

**Affiliations:** 1 School of Computing and Information University of Pittsburgh Pittsburgh, PA United States; 2 School of Pharmacy University of Pittsburgh Pittsburgh, PA United States

**Keywords:** sensing, medication adherence, active intervention, self-management, patient care, medication, qualitative study, successful intervention, patient support

## Abstract

**Background:**

Nearly half of Americans taking prescription medications do not take them properly. The resulting implications have a broad impact. Nonadhering patients develop worsened medical conditions and increased comorbidity of disease or die.

**Objective:**

Clinical studies have shown that the most effective strategies for addressing adherence are those that are individualized to the context that each patient and situation require. However, existing aids for adherence are relatively ridged and poorly support adaptation to individual behaviors and lifestyles. The aim of our study was to better understand this design tension.

**Methods:**

A series of 3 qualitative studies was conducted: a web-based survey of 200 Americans that investigated existing adherence strategies and behaviors and perception of how hypothetical in-home tracking technologies would assist adherence; in-person semistructured interviews with 20 medication takers from Pittsburgh, PA, that investigated personal adherence behaviors, which included demonstration of medication locations and routines as well as an assessment of hypothetical technologies; and semistructured interviews with 6 pharmacists and 3 family physicians to gain a provider perspective on patient adherence strategies, which included feedback on hypothetical technologies in the context of their patient populations. Inductive thematic coding of all interview data was performed. Studies were conducted consecutively, with the results informing the subsequent studies.

**Results:**

Synthesized, the studies identified key medication adherence behaviors amenable to technological interventions, distilled important home-sensing literacy considerations, and detailed critical privacy considerations. Specifically, 4 key insights were obtained: medication routines are heavily influenced and adapted by and through the physical location and placement of medications relative to activities of daily living, routines are chosen to be inconspicuous to maintain privacy, the value of provider-involved routines is motivated by a desire to build trust in shared decision-making, and the introduction of new technologies can create further burden on patients and providers.

**Conclusions:**

There is considerable potential to improve individual medication adherence by creating behavior-focused interventions that leverage emerging artificial intelligence (AI), machine learning (ML), and in-home Internet of Things (IoT) sensing technologies. However, success will be dependent on the technology’s ability to learn effectively and accurately from individual behaviors, needs, and routines and tailor interventions accordingly. Patient routines and attitudes toward adherence will likely affect the use of proactive (eg, AI-assistant routine modification) versus reactive (eg, notification of associated behaviors with missed dosages) intervention strategies. Successful technological interventions must support the detection and tracking of patient routines that can adjust to variations in patient location, schedule, independence, and habituation.

## Introduction

Despite the life-saving and life-preserving power of medications, it is estimated that nearly 50% of patients do not properly adhere to their medications [1]. The World Health Organization (WHO) has described nonadherence as a “worldwide problem of striking magnitude” [1]. Nonadherence leads to “worsening condition, increased comorbid diseases, increased health care costs, and death” [2]. Although adherent behaviors vary across pharmacotherapies and diagnoses [3], they are broadly defined by the US Food and Drug Administration (FDA) as “getting prescriptions filled, remembering to take medication on time, and understanding the directions as prescribed” [4].

Nonadherence behaviors are costly to national health care infrastructures and capacities. In the United States, US $100 billion in additional hospitalization costs and US $2000 per patient in physician visits per year are attributed to nonadherence [5]. These costs manifest across a wide variety of pharmacotherapy situations. For instance, in an acute situation of postoperative coronary artery bypass graft surgery, patients are placed on β-blockers and other medications to reduce occurrences of atrial fibrillation [6,7]. In the United States, adherence to these important postoperative medications is estimated to be <55% [8] and contributes to a postoperative readmission rate of 26.7% [9]. Similar situations occur with chronic conditions, too. Recent studies show that only 78.7% of insulin-dependent individuals with diabetes are adherent to ≥80% of their injections, increasing risk of stroke and other cardiac complications [10,11]; just 82% of patients who have undergone a kidney transplant are adherent to immunosuppressants that are critical to prevent organ rejection [12], and just 25% of patients prescribed pre-exposure prophylaxis (PrEP) are adherent to >90% of doses, increasing risk of contracting HIV [13].

Despite the scale of the problem, research and development efforts to build effective technology aids and interventions to improve medication adherence are quite small compared with the size of the consumer health care technology market. In part, this limitation is governed by the multitude of policy and economic factors that influence adherence, especially with access to medications. However, there have been evident studies that have shown that technology-based monitoring and reminders can provide sustainable improvement in medication literacy and adherence behaviors across a broad population of users [14-16]. However, these studies have also noted the highly individual situations and sentiments that define and motivate medication-related behaviors, noting that technology approaches cannot have ridged approaches to adherence [17,18]. These cited clinical studies have shown that utility is greatest in interventions that are highly customized to address a patient’s specific adherence barriers.

Poor efficacy and the use of common metrics or techniques across detection, classification, and intervention are consistent themes in previous efforts. For instance, Chung et al [19] observed that health-tracking applications fail because of the use of one-size-fits-all metrics rather than individualized health goals. Similarly, Clawson et al [20] found that health-tracking technologies were often abandoned when users failed to realize the benefits of the technologies, in part because of the failure of the technologies to support specifics of lifestyles and expectations. They continue to argue that health-tracking technologies must work in users’ complex social lives and highly individualistic activities of daily living. Complementing the challenges of technology are the limited insights from clinical perspectives to address adherence as a behavior. Clinical interventions include limited data from the health system and self-report [21]. Clinical practices are limited in their ability to understand, interpret, or adapt to the implementation of technology to support adherence.

The lessons of past technical and clinical work must guide future health technologies’ design and development, specifically the need to use methods that seek solutions that easily integrate and support highly specialized, personal behaviors. However, understanding the path to achieving this goal is still an active design and technology exploration. To this end, this paper presents a set of in-depth formative studies to evaluate the potential for new and novel medication adherence interventions across a variety of clinical needs, lifestyles, attitudes, and behaviors concerning medication and overall health. The results of our studies suggest that the needs of users are extraordinarily diverse, influenced by a multitude of factors, including daily schedules, number and type of medications, and level of overall health. More so, though, complex sociotechnical factors also influenced needs and perceptions of technology. The adoption of reminder technologies, for instance, was heavily tied to the perception of self-independence. Technologies that incorporate automation and behavior modeling raise concerns not just about privacy but also about the judgment of personal health behaviors. The work described in this paper contributes a series of descriptive insights and associated implications for the design of medication adherence tools.

## Methods

### Overview

A series of three qualitative studies was conducted as follows: (1) a web-based survey that investigated existing adherence strategies and behaviors and perception of how hypothetical in-home tracking technologies would assist adherence; (2) in-person semistructured interviews with medication takers from Pittsburgh, PA, that investigated personal adherence behaviors, which included demonstration of medication locations and routines as well as an assessment of hypothetical technologies; and (3) semistructured interviews with pharmacists and family physicians to gain a provider perspective on patient adherence strategies, which included feedback on hypothetical technologies in the context of their patient populations. Studies were conducted consecutively, with the results informing the subsequent studies. In each of the following subsections, we describe each study design and the evaluation methodologies used.

All 3 studies were designed to further understand how existing artificial intelligence (AI), machine learning (ML), and Internet of Things (IoT) technologies could be used to track, learn, and inform on adherence behaviors and potential interventions. Most prior work has focused on gaining population-level or intervention-level insights, which provide few practical design insights at the motivation and management levels. The methods of the 3 studies conducted in this work provide particularly useful investigations into how technologies could learn and leverage everyday routines and behaviors to drive interventions in adherence practice. These methods attempt to connect routines and behaviors with technology perception and acceptability.

### Formative Survey

We surveyed 200 people, all living in the United States, using a web-based 52-question survey (Multimedia Appendix 1). The questions were a mix of multiple-choice, short-answer, and free-form questions. It was deployed using Amazon’s Mechanical Turk (Amazon Web Services) and conducted over a 2-week period in late September 2019 [22]. All respondents were screened before completing the survey to be currently taking at least one prescription medication. Tables 1 and 2 report the number of medications per respondent and the age of the respondents, respectively. There were no interaction effects between the number of medications and age, although the number of medications trended upward with age. Respondents were most likely to be under the care of a single physician (105/200, 52.5%), although many were under the care of 2 (61/200, 30.5%) or 3 (20/200, 10%). The number of prescribing physicians (ie, respondents had an active prescription from this physician) was similar; most had only 1 (132/200, 66%), followed by 2 (44/200, 22%) and 3 (6/200, 3%). Each respondent was paid US $4 for completing the survey. The mean completion time was 9 minutes, 42 seconds (SD 5 minutes, 31 seconds).

**Table 1:** Number of medications taken by survey respondents (N=200).

Number of medications taken	Respondents, n (%)
1	71 (35.5)
2	54 (27)
3	36 (18)
4	15 (7.5)
5	12 (6)
≥6	12 (6)

**Table 2:** Age of survey respondents (N=200).

Age range (years)	Respondents, n (%)
18-24	6 (3)
25-34	55 (27.5)
35-44	61 (30.5)
45-54	35 (17.5)
55-64	25 (12.5)
≥65	18 (9)

### In-Home Interviews With Medication Takers

A combination of in-person and remote video conference semistructured interviews was conducted with 20 medication-taking participants between February 2020 and September 2020. A total of 50% (10/20) of the interviews were conducted in person, and 50% (10/20) were conducted using videoconference. The in-home interviews were recorded to capture audio and video. This method also allowed for the observation of described adherence behavior within the context of broader home activities, medication location within the physical home layout, and second-party (eg, spouse) participation in routines. While in the middle of conducting our interviews, the COVID-19 pandemic prevented in-person human participant studies at our institution; thus, 50% (10/20) of the participants were interviewed using a Health Insurance Portability and Accountability Act of 1996 (HIPAA)–compliant videoconferencing software. The sample size was based on previous health behavior studies and broader guidance across the discipline [23].

The interviews were conducted using a semistructured protocol. Guiding questions were used to explore interviewees’ perceptions of health status, current disease states and medications, medication adherence and daily personal routine, use of medication adherence tools, use of digital tools to support health and routine use in the home, and concerns about using technology to track and manage health. Participants were also asked to either draw a diagram of their home or describe their residence and describe the relationship between the participant and any other cohabitants and how, if at all, any cohabitants assisted in medication adherence or other experiences in health care. These guiding questions were followed by probing questions based on participants’ responses. The interviews ended with a series of structured feedback responses. Participants were provided with scenarios that described the same hypothetical technologies used in the formative survey. Interviewees provided a response as to whether each proposed technology would be *very useful*, *somewhat useful*, or *not useful*. We purposely used a less structured scale compared with the survey to facilitate being able to pivot the discussion to ask the participant to explain their rating. The full semistructured interview protocol is provided in Multimedia Appendix 2.

Medication takers were recruited using our university’s clinical research registry. Participants were recruited from a pool of registrants with a known diagnosis of hypertension and currently taking one or more prescription medications. The use of this hypertensive pool was a convenance for recruiting—these studies and their results are very likely to represent a broad population of medication takers. The pool itself comprises many patients across a broad set of sociodemographic categorizations. All participants were cognitively competent adults from the Pittsburgh region, with ages ranging from 27 to 71 (mean 47.75, SD 15.35; median 43) years. A total of 70% (14/20) identified as female, and 30% (6/20) identified as male. Participants reported the total number of prescription medications taken; the number ranged from 2 to 13 (mean 5.79, SD 3.65; median 5). The interviews lasted between 90 and 120 minutes. Each participant was compensated with US $40 for taking part in the study.

Interview recordings were outlined using the interviewers’ notes and transcribed to identify quotes. Interview transcriptions and notes were analyzed using a general inductive approach by 4 researchers. General topics were coded (eg, location in which the medications were kept, current medical conditions, and relationship with cohabitants). Cross-coding was performed on a random sample of 30% (6/20) of the interviews. Once coded, the same 4 investigators engaged in a systematic review of the coded interviews to organize them into categorical findings.

### Interviews With Health Care Providers

Health care provider interviews were conducted after the medication taker interviews were completed. These were conducted at the location of practice (retail pharmacy, medical practice, or hospital) or via teleconference to accommodate the provider’s comfort with in-person interaction during the COVID-19 pandemic. A total of 9 interviews were conducted: 6 (67%) with pharmacists and 3 (33%) with family practice physicians. The interviews were conducted between July 2021 and August 2021. Owing to the institutional regulations of interviewing providers and associated clients, we did not seek to interview providers of the medication takers interviewed. We recruited providers through snowball sampling within the authors’ professional networks. Participants were from several organizations, including family practices, clinic pharmacists, and community pharmacists. The sample size was based on broader guidance across the discipline [23] and guidelines for expert interviews [24].

A semistructured interview protocol was used. The interviews focused on 3 main points of inquiry: overall concern with clients’ medication adherence behaviors and compliance, provider prospective of adherence obstacles and assessment of the mitigating context, and exploring existing methods used to promote adherence and the limitations of those approaches. As we recruited patients with hypertension as medication takers, we asked providers to answer questions specifically about patients with hypertension, although they would often indicate that responses applied more broadly to their client populations. Providers were presented with scenarios that described hypothetical technologies. They were asked to describe how useful they felt these technologies were overall and provide insights into the type of clients they felt would be more and less receptive to using them. We did not collect the age of the providers; all had >10 years of practice in their roles. The interviews lasted approximately 60 minutes. Participants were compensated with food or a gift card for lunch at a local restaurant. We used the same analysis techniques and cross-coding procedures used for the medication taker interview data. The full semistructured interview protocol is provided in Multimedia Appendix 3.

### Ethics Approval

Each study was reviewed and approved by the University of Pittsburgh institutional review board (STUDY19080322, with protocol title “Technology for Prescription Adherence”).

## Results

The 3 studies were performed sequentially; each study was informed by the previous one. Thus, we present the results sequentially.

### Findings From the Formative Survey

The formative survey provided insights into broad adherence practices, existing technology use, and sentiment on potential technology aids. Common behaviors associated with missed dosages were indicated and rated for frequency (daily, weekly, monthly, and yearly). The reasons were presented as a list of behaviors derived from a synthesis of previous studies. Responses are summarized in Table 3. A Pearson contingency analysis indicates that a significant difference was observed across groupings by reason (χ^2^_42_=419.1; *P*<.001).

Respondents were asked to rate their use of commonly recommended and prescribed adherence aids, namely, pill box or medication organizer and diary or calendar. Indications and frequency of use were also measured. As summarized in Table 4, the means trended positively with frequency of use. A Kruskal-Wallis test on differences in the effectiveness ratings (grouped by use) was significant for both the pill box (χ^2^_3_=60.5; *P*<.001) and diary (χ^2^_3_=15.0; *P*<.002) aid types. Only 14% (28/200) of the respondents indicated use of an existing medication-tracking digital application. Ranking the top features, of these 28 respondents, 14 (50%) indicated reminders and notifications, 6 (21%) indicated the ability to track medication doses, and 4 (14%) indicated signaling the pharmacy to refill the prescription. Of the 172 respondents not using an application, 61 (35.5%) responded that the use of a smartphone for tracking medications was too cumbersome, 47 (27.3%) expressed concerns about privacy and security, and 27 (15.7%) indicated that they did not use their smartphone in the same location in which they stored and took their medications. Respondents stored medications in a variety of locations: the bedroom (80/200, 40%), the kitchen (74/200, 37%), and the bathroom (41/200, 20.5%). A total of 16% (32/200) carried medications on their person. Many (50/200, 25%) indicated that they kept medications in more than one location in their home, but only 1% (2/200) indicated keeping medications outside their home.

Respondents were given brief descriptions of 6 hypothetical smart home technologies. These technology descriptions were based on generic approximations of technologies that could be imagined based on current state-of-the-art IoT technologies. The specific descriptions are provided in Textbox 1. Respondents were asked to “rate the perceived utility of [the] technology.” Respondents were then asked to rate whether the technology would be “more or less useful than their current medication adherence practices*.*” Table 5 summarizes respondents’ feedback. The results confirmed that adherence remains difficult for a broad set of American adults, young and old. Across failures to adhere, unintentional reasons (eg, forgetting the dose and being away from the medication) were more frequent than intentional reasons (eg, avoiding side effects and having no symptoms). A common theme among respondents was that there were *multiple* reasons for missed doses, reflecting the complexity and deep embedding of adherence behaviors within daily activities and routines. The usefulness ratings of the hypothetical technologies strongly connected with perceived use in improving adherence. These results motivate the need for technologies that reinforce habituation, have low barriers to use, and adapt to the complexity of medication storage and interaction.

These results were void of the context necessary to understand how and why people choose their medication adherence routines, how they use their selected tools and aids to support those routines, and the factors surrounding the daily challenges of medication adherence. These in-depth questions necessitated and informed the design of a qualitative approach based on in-depth interviews with patients and health care providers. In study 2, we interviewed patients. In study 3, we interviewed health care providers.

**Table 3:** Respondent frequency of reasons for missing medication doses (N=200).

Reason	Missed dose at least once per..., n (%)			
	Year	Month	Week	Day
Medication dose forgotten	136 (68)	88 (44)	23 (11.5)	1 (0.5)
Medication in a different location	106 (53)	58 (29)	13 (6.5)	1 (0.5)
Activity prevented taking medication	85 (42.5)	47 (23.5)	16 (8)	2 (1)
Medication supply ran out	51 (25.5)	11 (5.5)	2 (1)	1 (0.5)
Avoidance of side effects	47 (23.5)	25 (12.5)	14 (7)	4 (2)
No symptoms present	43 (21.5)	26 (13)	11 (5.5)	2 (1)
Making the medication last longer	43 (21.5)	23 (11.5)	8 (4)	1 (0.5)
Social situation made it inappropriate	34 (17)	17 (8.5)	8 (4)	0 (0)

**Table 4:** Perceived effectiveness of existing aid by frequency of use (N=200).

Frequency of use	Pill box				Diary		
	Participants, n (%)	Rating, mean (SD)	Post hoc	Participants, n (%)		Rating, mean (SD)	Post hoc
Always	44 (22)	4.66 (0.71)	<.001, approximately half of the time; <.001, sometimes	7 (3.5)		4.43 (0.79)	.01, sometimes
Most of the time	23 (11.5)	4.17 (0.49)	.002, sometimes; <.001, always	10 (5)		4.10 (0.74)	.03, sometimes
Approximately half of the time	10 (5)	3.20 (0.79)	<.001, always	4 (2)		3.00 (0.82)	N/A^a^
Sometimes	33 (16.5)	3.12 (0.82)	.002, most of the time; <.001, always	29 (14.5)		2.93 (1.14)	.01, always; .03, most of the time

^a^N/A: not applicable.

**Description of the hypothetical technologies used in the formative survey.**

Proximity Notifications (PN)

- A technology that would detect and alert when you are near your medications, providing in-situ notifications for scheduled doses.

Proximity Notifications to Caregiver (PN-Care)

- A technology that would detect and alert when a caregiver, family member, or trusted aide is near your medications.

Pre-Departure Reminder (PDR)

- A technology that would detect and alert when you are about to leave your home without taking scheduled medications.

Routine Change Detection (RCS)

- A technology that would learn more about your behaviors and movements when you are within and away from your home. It could use this information to suggest times and locations for taking medications that could lead to improved adherence.

Routine Change Detection Supported with Healthcare Providers (RCS-Team)

- A technology that would learn more about your behaviors and movements when you are within and away from your home. Behaviors and movements would be summarized and made available to your healthcare professionals. These summaries could be used to improve medication selection, scheduling, dosing, and other instructions provided by your healthcare professionals to improve adherence.

Medication with Food (MwF)

- A wearable or smart home technology that would learn more about your behaviors to classify when you are eating a meal. The technology could help remind you to take medications that need to be taken with a meal or simply help establish a routine of taking medications with meals.

**Table 5:** Perceived usefulness and impact on existing personal adherence for each hypothetical technology (N=200).

Hypothetical technology and usefulness rating			Participants per usefulness rating, n (%)	Perceived impact on existing adherence regimen, n (%)								
				Much worse		Somewhat worse		Same		Somewhat better		Much better
**PN^a^**												
	Very	53 (26.5)		0 (0)	2 (3.8)		25 (47.2)		11 (20.1)		15 (28.3)	
	Somewhat	96 (48)		8 (8.3)	40 (61.7)		45 (46.9)		3 (3.1)		0 (0)	
	Not	51 (25.5)		22 (43.1)	24 (47.1)		5 (9.8)		0 (0)		0 (0)	
**PN-Care^b^**												
	Very	29 (14.5)		0 (0)	0 (0)		5 (17.2)		8 (27.6)		16 (55.2)	
	Somewhat	61 (30.5)		0 (0)	3 (4.9)		36 (59)		17 (27.9)		5 (8.1)	
	Not	110 (55)		32 (29.1)	26 (23.6)		49 (44.5)		1 (0.9)		2 (1.8)	
**PDR^c^**												
	Very	96 (48)		0 (0)	0 (0)		5 (5.2)		34 (35.8)		56 (58.9)	
	Somewhat	71 (35.5)		2 (2.8)	3 (4.2)		19 (26.8)		44 (62)		3 (4.2)	
	Not	34 (17)		11 (32.8)	5 (14.7)		17 (50)		1 (2.9)		0 (0)	
**RCS^d^**												
	Very	38 (19)		0 (0)	0 (0)		1 (2.6)		18 (47.4)		19 (50)	
	Somewhat	71 (35.5)		0 (0)	3 (3.4)		39 (44.3)		39 (44.3)		7 (8)	
	Not	74 (37)		27 (26.4)	16 (21.6)		31 (41.9)		0 (0)		0 (0)	
**RCS-Team^e^**												
	Very	27 (13.5)		0 (0)	0 (0)		0 (0)		9 (33.3)		18 (66.6)	
	Somewhat	78 (39)		0 (0)	2 (2.5)		34 (44.3)		38 (48.7)		4 (5.1)	
	Not	95 (47.5)		34 (35.8)	24 (25.3)		35 (36.8)		2 (2.1)		0 (0)	
**MwF^f^**												
	Very	60 (30)		0 (0)	0 (0)		3 (5)		23 (38.3)		34 (56.6)	
	Somewhat	85 (42.5)		0 (0)	1 (1.2)		34 (40)		46 (54.1)		4 (4.7)	
	Not	55 (27.5)		12 (21.8)	11 (20)		30 (54.5)		2 (3.6)		0 (0)	

^a^PN: Proximity Notification.

^b^PN-Care: Proximity Notifications to Caregiver.

^c^PDR: Pre-Departure Reminder.

^d^RCS: Routine Change Detection.

^e^RCS-Team: Routine Change Detection Supported with Healthcare Providers.

^f^MwF: medication with food.

### Findings From the In-Home Interviews With Medication Takers

#### Overview

Survey responses did not have adequate context to fully understand how the environment and living configurations of respondents affected their medication adherence routines, how they used their selected tools and aids to support those routines, and the factors surrounding the daily challenges of medication adherence. These in-depth interviews provided a deeper design understanding based on the broader understanding provided by the formative survey. The interviews provided considerable qualitative insights into the causes of missed dosages and the use of medication adherence aids, as well as usefulness and utility insights for the hypothetical technologies.

#### Location and Routine as an Adherence Aid

The interviews found the location and placement of medication to be a key component of medication adherence behavior. All interviewed medication takers (20/20, 100%) provided very detailed explanations and rationales for where medications were kept. The desire to position medications so that they were near and accessible during activities of daily living was the most substantial motivation (17/20, 85%). Most interviewees placed their medications in locations that were proximal to where morning and nighttime activities were performed. For instance, medication taker 17 kept her medication on her nightstand next to her glasses. Describing her morning behaviors, she explained that she “grabs glasses and pills in the morning when waking up, takes pills to bathroom to take first thing.” Medication taker 19 kept her medication on top of her refrigerator, using it as a visual cue to take medications as part of her “breakfast and coffee routine.” Some chose different locations based on medication type (eg, medication taker 2 stated that “I keep my pills in the nightstand, blood sugar stuff by the TV, and my asthma stuff on the dresser”), and others did so based on the time of day the medication was taken (eg, medication taker 11 said that “the ones I take in the morning I keep in a blue basket on top of the microwave, the ones I take at night I keep in the nightstand next to the bed”).

A second placement rationale focused on keeping medications away or out of reach from young children (3/20, 15%). For instance, medication taker 1 indicated that she would place her medications on the top shelf of her closet when her grandchildren visited. Medication taker 3 similarly indicated placing her medications in a drawer “out of sight” when her grandchildren visited. These locations were often a compromise or completely against rationales of placement around daily living activities. For instance, medication taker 19 noted that she used to keep her medication in a kitchen cabinet but once found her child standing over the sink holding the medications and noted that it was a “not going to happen anymore” moment.

Interestingly, the locations were highly individual to each person, with no single rationale emerging as a general trend. Participants recognized in many cases the idiosyncrasies of their rationale. For instance, medication taker 20 kept the bottle for his pills in an ottoman in his bedroom, noting when explaining that “I know this sounds weird, but it works for me.” Medication taker 1 kept her pills in an old cookie tin and carried it around with her from room to room in her home:

At one time, I was a scatter brain, my meds would be all over the place. “Where’s this? Where’s that?” I said, “I can’t live like this.” Everything has to be in order. When everything is scattered, I’m out of control.

#### Adherence Behaviors Are Private

Medication management behaviors, including adherence, were highly private activities for most interviewees. A total of 75% (15/20) of the participants lived with someone whom they felt was a “trusting relationship,” but only 27% (4/15) indicated that their cohabitant partner assisted in adherence-related behavior. This was similar to the number of participants (5/15, 33%) who indicated that their trusted partner could communicate with medical providers on their behalf. We found that this privacy was centered on concerns of independence and perceived burden. For instance, when medication taker 4 was asked if his wife helped with his adherence, he responded with “no, and I don’t wear Velcro shoes either,” insinuating that, in addition to managing his own medication adherence, he was also able to put his shoes on independently. Similarly, medication taker 14 stated:

There are certain things as a grown up that you do by yourself.

For those who did receive help from trusted partners or cohabitants, it was usually motivated by their medical condition having a dramatic impact on their ability to independently perform activities of daily living. For instance, medication taker 6 had an injury preventing him from leaving his bed. A few of the older medication takers interviewed also indicated an openness to receiving help when they could not manage adherence on their own. For instance, medication taker 11 stated the following:

If I needed help, I wouldn’t be afraid to ask, I just don’t need help yet.

The private nature of medication behaviors was also shown in interviewees’ concerns about interventions that involved other individuals. When asked about smart speaker reminders for medication, medication taker 17 indicated that she was hesitant to use her Google Home for personal health as “if [she] had friends over, [she] would not it want to announce.” Independence concerns were also noted in the hypothetical technology ratings, with only 30% (6/20) of the interviewees indicating that they found Proximity Notifications to Caregiver (PN-Care) to be very useful, whereas most (12/20, 60%) found it not useful.

#### Role of Health Care Providers in Medication Adherence

Involving health care providers in medication adherence tools elicited mixed opinions. Many felt that it would be helpful in establishing trust by relaying information about adherence behaviors to physicians. In total, 55% (11/20) of the medication takers indicated that Routine Change Detection Supported with Healthcare Providers (RCS-Team) would be very useful, 25% (5/20) indicated that it would be somewhat useful, and 20% (4/20) indicated that it would be not useful. For those indicating usefulness, the motivation was primarily centered on establishing a stronger understanding of medication behavior and effectiveness with the provider. For instance, medication taker 14 stated the following:

I would like the doctor to have a more factual set of information for I’m doing as opposed to pre-conceived notions that they have.

Medication taker 15 stated that the hypothetical technology “would be very helpful if it would help with adjustments of something not known to self*,*” indicating that health care providers may use it to improve diagnosis or treatment.

Medication takers expressed concerns about the reach of technology; in particular, concerns about privacy and the negative impact on care were expressed. Medication taker 11 was very articulate in her concerns:

There’s a thin line there with how much your doctor needs to know. I guess it would depend at what you’re looking at. If it were to cure cancer, I’d say it’s very useful, but if it were for something minor, I wouldn’t be for it.

Medication takers felt that it was simply unnecessary for the physician-patient relationship. Medication taker 12 relayed the following:

If I want to express something to my health care provider, I would just tell them, I don’t need an app to do it.

#### Role of Technology in Health

Privacy concerns with the hypothetical technologies were mixed. Medication taker 1 was very split on achieving the benefits at the cost of privacy:

I heard [about this] technology that learns your behaviors. I mean it’s scary to give technology that much power. I don’t know what to do. I’m for it to a certain degree. It’s good that it does learn your behavior, but it’s too much.

Medication taker 12 provided a more succinct value proposition:

Security is always a good thing to keep in mind, I would really have to want a technology to get it.

Other medication takers expressed less concern, focusing on the perceived low utility of their personal health information to others. Medication taker 11 stated the following:

I don’t worry too much about my privacy. I have a mindset that I don’t have that much to hide, if you want to know I don’t care.

Medication taker 8 echoed a similar sentiment—“To be honest, I’m an open book. information like that doesn’t bother me at all”—referring to personal medical history. Overall, we found the lack of privacy concerns to be a somewhat stark contrast to the private nature of medication adherence.

### Findings From Interviews With Health Care Providers

The provider interviews were an important complement to the medication taker interviews. To allow for a straightforward comparison, we organized the results in the same structure as the findings from the medication taker interviews.

#### Location and Routine as an Adherence Aid

The effectiveness of routines and aids was more sobering from the perspective of pharmacists and physicians. In our interviews, we quoted the literature that indicated that <50% of patients overall are adherent; when asked if they agreed, all providers (9/9, 100%) responded with overwhelming agreement. When asked to describe the reasons for nonadherence, physician 2 noted that most nonadherence behaviors are not intentional:

In my [patients] it tends to be more ability to pick up medicines, ability to be organized enough to manage the medicines, that sort of thing. More so than their overt, “I’m not going to take this.”

Physician 3 noted similar reasons and was empathetic to the patients:

I mean, I think they all try to...it’s that they forget they fall asleep at night, so they forget their nighttime dosage. For most people, I think they want to be compliant. It is a matter of when you don’t have a symptom. How do you remember to take medication every day?

The most common mechanism providers have to monitor adherence is refill statistics, which are commonly available to both pharmacists and primary care physicians. They noted that refill cadence often does not match the prescription, indicating that doses have been missed. Providers often discuss this with clients, as pharmacist 2 stated:

I go through the normal questions, are you having side effects? Let’s talk to your doctor about lowering doses. There’s a reason you’re not taking it. Are you having side effects? [Do you] just forget to take [it]?

Physician 1 provided a perspective that emphasized the prevalence of nonadherence but also noted the current limitations in their ability to understand client adherence behaviors:

I think that their compliance, because I have written an order; they say that they’re engaged. [But] we don’t really understand the level of non-compliance. And, therefore, to put that [effort] into [an] understand why this [happens] is hard.

This was echoed by pharmacist 4:

I definitely [try] to give them benefit of the doubt, but I feel like even after I go through all of those questions and the patient’s like no I take every day, no problems, no issues. I think if they could just be honest with us and help us, to help us identify those adherence issues and we can help them solve it.

Prepackaged medications received mixed reviews from the health care providers interviewed. Providers indicated that this approach works well for patients who lack home health assistance either from a family member or outside organization. Providers also pointed out a considerable limitation: a prepackaged medication routine can quickly become confusing and difficult for patients to manage when medications or dosages are changed. Physician 2 stated the following:

...anytime you make an adjustment, it’s a delay in them getting the new meds so and then it’s sort of a rigmarole to get them changed. And I’ll have patients that ended up with other meds that are now in separate bottles. And so, somebody has to be orchestrating the pill packaging.

Providers were supportive of pill organizers, and they indicated that they often recommended that patients use them. Physician 2 noted that she had pill organizers at her clinic and would, on occasion, even demonstrate to patients how to fill them. Interestingly, providers often noted that using an organizer alone is not sufficient; providers stated that it also matters where the pill organizer is kept. Physician 3 recalled a recent conversation about the location of the pill organizer:

He has [his medications] beside his bedside table. But he falls asleep and doesn’t remember.

Providers were aware of the diversity of behaviors regarding where their clients kept their medication. Physician 3 noted that she would often review the location of her clients’ medications as part of adherence discussions. Providers indicated that they often had to review medication storage locations as living situations change and can disrupt adherence routines. For instance, physician 1 recalled several conversations with his low-adherence patients and noted the following:

It’s one of the things we find often is the medications in the bedroom, or it’s in the living room, and [they say they] spend all the time in the bedroom in the summer, because that’s for the air conditioner, or something.

#### Adherence Behaviors Are Private

Privacy and independence concerns between a medication taker and a provider rarely exist. This mirrors the results from the patient interviews and is perhaps not surprising considering that physicians and pharmacists are trusted individuals who prescribe and dispense the medications. Providers were asked to reflect on how their clients rely on family members and cohabitants for adherence. The responses were consistent with those of medication takers. Family and friends were used for transportation to the physician’s office and pharmacy or were asked to pick up refills. Physicians also noted that it was very rare for clients to have others present in the examination room. Physician 2 noted the following:

I would say probably about 5 or 10% of my patients come with somebody.

#### Role of Health Care Providers in Medication Adherence

Providers expressed great interest in having tools that could help them understand client medication adherence behavior. Pharmacist 1 expressed interest in getting a sense of overall adherence trends, noting the following:

...if [patients] reported on a weekly basis of like how many days that they missed, or what days that they missed their medications, it would be super helpful.

Physician 1 went further, indicating that more information would have actionable value, stating the following:

...system, and it says, you know, [patient name] took his medicine two days out of 10 this week, or 10 times in the last three days, that’s very helpful information. Because if the system allows for health care proxy kind of function, then that’s, that’s a level of sort of monitoring compliance, that could be very helpful. That doesn’t involve us or could escalate, you know, hey, [patient name] is really not taking this medicine. What else can we do here? Can we change the system?

In many ways, this was similar to comments from medication takers, who wanted to use data to force action or attention from a care provider. Similarly, providers also mentioned concerns about privacy. For instance, when physician 2 was asked for feedback on RCS-Team, she stated the following:

It seems like a cool idea. But sort of invasive [of] patients with privacy issues, it seems like big brother’s watching, I don’t like it.

#### Role of Technology in Health

Provider feedback on technology opportunities was overly focused on the limited time they had with patients and worries that introducing a new technology would take time away from other important tasks. When asked about reviewing patient data in the context of RCS-Team, physician 2 stated the following:

...would I have time? My immediate answer is no, of course not. I don’t have time to do what I need to do already. What am I skipping to add that in? There is no time to do anything extra. Unless somehow miraculously, it helps with something else so that it slims down, you know, tears down the rest of the junk I have to do.

In addition, providers were concerned about the burden they would feel to make the data actionable. Physician 1 expressed an articulate vision of the long-term insights that technology could provide into patient health but struggled with how he could expect that exploring data and formulating insights into a useful recommendation to patients would fit into his busy work practice:

[to know] their blood pressure’s normal 85% of the time and they’re taking their medicine 87% of the time and [the medication is kept] at the front door, often, maybe that part could get us to 90% of the time, like, you know, something that would sort of look at this a little intelligently and say, so the problems not necessarily compliance, but with that 13% compliance. It just needs to tell me where the opportunities are.

## Discussion

### Principal Findings

These studies indicated several key insights to better leverage technologies to support challenges with medication adherence. Similar to the adherence challenge itself, there were many complicated sociotechnical concerns raised that will challenge the design of these future technologies. These range broadly from the diversity of each individual’s adherence behaviors and routines to the complications of designing technology that can be flexible to support a diverse set of needs at a low cost. In particular, the analysis uncovered the need for technologies that do not just target one behavior or action but are broad and flexible enough to achieve sustained utility as needs and practices shift.

### Strengths and Limitations

This study contributes an understanding of the perceived use of technology-aided medication adherence tools and interventions. Although current approaches to medication adherence can implement static routines or idealized behavior, this study can inform the development of sensing, persuasive, and other technologies that meaningfully leverage users’ current behaviors and adapt to changing needs. The most substantial limitation is that the collected user sentiments were based on previous behavior, preference, and perception of hypothetical technologies, not on actual use. The formative survey was limited to 1 Western-culture country, and the interviews were focused on a specific geographic region of that country. Future investigations are needed to understand how cultures, access to health care, governmental influence, and many other factors affect adherence behaviors and adherence technology aids. Our investigation also focused on medication takers, pharmacists, and primary care physicians. It did not include other important entities such as health care system administrators or operators, insurers, or government aid agencies. Future investigations with these other stakeholders will be needed to fully translate design insights into actionable technologies for changes in practice and outcomes [25].

### Comparison With Prior Work

Most medication adherence studies have focused not on intervention assessment but on the clinical outcomes of adherence within particular populations and diagnoses [26]. These studies can be epidemiological in focus or more targeted in scope, for instance, clinical trials of new medications where adherence is a requisite for measuring efficacy. Across studies, methods for collecting adherence data are surprisingly manual and analog. Such studies, their methods, and their results do not provide deep insights into adherence behaviors.

Distilling poor adherence to a specific behavior or factor is not possible [27]. Unintentional patient behaviors are understood to be a substantial contributor to nonadherence [17]. For instance, a recent study on patients with heart failure found that nearly 50% of missed doses were attributed to forgetfulness [28], matching the results from the studies presented in this paper. Interventions to address unintentional nonadherence have not had great success. A large study comprising 53,480 patients and 2 years of prescription data showed that low-cost reminder devices did not improve adherence [29]. Analyses of interventions have found that “to improve adherence effectively, there is a need for a tailored approach based on the type and cause of nonadherence and the specific needs of the patient” [17]. Recent surveys of adherence studies further support the need for tailored aids that allow for effective counseling and feedback, both automated and provider generated [30,31]. Bateman [32], reflecting on study adherence aids for patients with asthma, noted the need for “customized patient-friendly treatment that anticipates and accommodates usual behavior…is more likely to achieve the desired goal of disease control*.*” We believe that our studies provide new design insights toward meeting this goal.

Existing technology-based adherence intervention research has established that personalized interventions are essential for technologies to be efficacious [14,33]. Previous work has also illustrated that this level of personalization is difficult using traditional technologies (eg, smartphone apps) [14]. Other work has established the need for technologies to leverage known behaviors around routines, especially those specific to space use and medication storage [34-38]. The studies by Palen and Aaløkke [33] and by Dalgaard et al [39] have shown the importance of technologies that involve care providers, particularly in relation to patient-provider interactions. Our work builds on these past works; we advanced the design understanding of how technologies can be used to drive personalized interventions that leverage a combination of behavior sensing and models of effective adherence and self-monitoring.

### Implications for Practice, Research, and Design

#### Overview

These 3 studies provided several key insights to better leverage technologies to support challenges with medication adherence. Similar to the adherence challenge itself, there were many complicated sociotechnical concerns raised that will challenge the design of these future technologies. These range broadly from the diversity of each individual’s adherence behaviors and routines to the complications of designing technology that can be flexible to support a diverse set of needs at a low cost. In particular, the analysis uncovered the need for technologies that are broad and flexible enough to achieve sustained utility as needs and practices shift.

#### Routine-Aware Aids

One of the most striking adherence behaviors uncovered was the extensive organizational routines that punctuate activities of daily living, for instance, placing medicine on nightstands to be the first point of attention in the morning and flipping medicine bottles over once taken in the morning. Most of these behaviors were adopted because they allowed the repetitive, routine nature of daily medication activities to be, as explained by medication taker interviewees, difficult to ignore, skip, or alter. Further, we found that pharmacists and primary care physicians encourage their clients to adopt these behaviors and even spend time with patients to discuss and strategize these efforts. In contrast, our survey found very few people who used technology reminders or alarms to support medication adherence routines; in fact, our interviews found only 1 medication taker who used an alarm on their smartphone. Alarms, along with existing smartphone app–based medication adherence tools, lack activity context. Specifically, alarms and apps will present reminders *when* medications should be taken, not *when and where* they should be taken. As a medication taker interviewee stated, an alarm going off when one is away from one’s medication is a “useless” reminder; it would simply be dismissed as taking the medication would not be possible or convenient at that moment. This implication raises serious concerns about the sustainability of adherence behavior change interventions documented in the adherence randomized clinical trial literature.

In contrast to the lack of perceived usefulness of alarms, the presented hypothetical Proximity Notification (PN) was perceived as very or somewhat useful (150/200, 75% in the survey and 13/20, 65% in the interview). We believe that this large difference is a result of the proximity-based notification’s ability to capture the *where* component of medication adherence behaviors. Furthermore, in our formative survey, we asked respondents to rate the usefulness and perceived impact on medication adherence behaviors. The ratings were highly related: the higher the usefulness, the higher the perceived impact. On the surface, this relationship is not surprising, but it explains a broader expectation that potential users of technologies need to understand and appreciate the impact on *their* behaviors and routines. Indeed, when speaking with providers, patients who were able to make this connection in their behaviors were the most successful with adherence. In fact, many health maintenance routines have similar *when and where* routine-driven use, which suggests that design for this time or place context needs to be more deeply considered in the development of future technology aids.

#### Aids Not Just for “Every Day” but for All Days

Previous research has shown that technology adoption and use are *messy* in the real world. Clawson et al [20] concluded that self-monitoring health technologies need to be designed to accommodate the “ever changing dynamics of individuals’ lives.” Our investigations suggest that there is a need to address dynamics that *are not* changing over time but that are a part of the regular *messiness* of activities of daily living. Unsurprisingly, we found that routines vary from one day to the next. One day might be a trip to church, another might involve volunteering at the community center, and yet another might involve sports activities. These daily dynamics wreak havoc on a person’s ability to follow a tight daily routine, punctuated by small repetitive behaviors such as taking medications. Further, providers noted that changes in dosages or additions of medications were *danger zones* for nonadherence as they involved a routine change. Particularly troublesome were the times of transition in care (eg, being discharged from inpatient care).

Future technologies for health interventions will need to use adaptive aids to accommodate the dynamics in these activities of daily living or will need to be agnostic to the daily dynamics. For instance, medication takers in our studies suggested that tools that can react to these changes were perceived as useful. Specifically, ratings on Pre-Departure Reminders (PDRs) were high (166/200, 83% in the surveys and 13/20, 65% in the interviews), as were the ratings on perceived impact (Table 5). Similarly, reminders centered on mealtime also had considerable usefulness ratings (146/200, 73% in the surveys and 13/20, 65% in the interviews) and potential to affect adherence behaviors. Managing these types of dynamic aids, particularly how they can adapt to day-to-day and week-to-week variations in a scalable manner, engage with individuals and their personal context and circumstances, and effect positive changes via personalized and context-driven interventions, remains a substantial challenge.

#### Reactive Versus Proactive Aids

Our combined studies provided an interesting context to understand the broader expectations of intervention functionality for health-focused technologies. Although it was not surprising to confirm differing opinions from potential users (and even differences in opinions among the care providers), it was interesting to observe a divide between medication takers who preferred aids that were reactive in their design (eg, the PN and PDR notification hypothetical technologies) and those who preferred aids that were proactive in their design (eg, the Routine Change Detection [RCS] and RCS-Team hypothetical technologies). When asking medication takers to contextualize their preferences, it became apparent that there were fundamental differences in the motivation for these preferences. In particular, reactive aids were strongly preferred by medication takers who preferred technologies to, as a medication taker said, “help me be me.” These were medication takers who had clear, well-defined daily medication routines; had high personal motivation to maintain or improve their health; and (most importantly) did not feel the need for a technology recommending or forcing the adoption of new routines or changes to daily activities. In contrast, we had medication takers who clearly wanted the technologies to “help me be a better me” (adaptation of the previous quote). These interviewees were less organized in their routines or simply overwhelmed by their medication regimens or activities of daily living. Simply put, they were looking for aids that could provide answers and insights to make their lives better.

Indeed, medication takers wanted the technologies’ proactiveness to extend to health care providers, initiating changes in dosage or medication selection. Providers were also interested in leveraging these technologies; however, they were greatly hesitant to embrace any technology that would be an additional responsibility (and time commitment) for their professional practice. Future technologies must be designed to recognize the preferences and needs of the individual medication taker. Ideally, technologies should also create pathways to bridge their users from one intervention strategy to another. The technologies should bring more independence and confidence to those who can begin to master their personal health and provide increasing support to those who struggle, perhaps through strategic data sharing and the involvement of a medication taker’s pharmacist and primary care physician.

#### Aids to Promote Independence

A common sentiment of concern expressed by patients was the perception of their independence in their own care. We found a strong aversion to enabling other people to participate in their medication adherence activities and behaviors. Even in the case of medication takers who lived with cohabitants, most of whom were also intimate partners, they were nonetheless not included in activities related to their personal health. Pregnancy and debilitating conditions were the only exceptions, and still, independence was guarded. These sentiments carried over to technology perception. Our lowest-rated hypothetical technology was one that explicitly involved other cohabitants in medication adherence behaviors. Further, a concern expressed regarding smart speakers such as Amazon’s Alexa was that they would share these private behaviors with the household. This leads to an important design lesson: health technology aids must allow users to feel *and* be in control, assuring them that they are independent and not dependent on others or even the technology itself. It is important to note that autonomy is not just a design goal but also a clinical goal [40]. Aids that involve or promote engagement with health care providers can improve overall health care utility and use. Technologies that can represent a strong connection between patients and health care providers can capitalize on this underrealized potential. As evident in our conversations with providers, there was a strong desire for technologies that could empower patients to better understand and take control of their health care.

### Conclusions

Adherence to prescription medications is a ubiquitous and nuanced challenge for many people. Technology offers a promising contribution to this challenge based on its ability to learn from individual behaviors, needs, and routines and tailor interventions accordingly. Our formative survey results reaffirmed the challenges that people face when attempting to be adherent to their medications and characterized the relationship between technology and users’ needs, motivations for use, and expectations. Our interviews with medication takers and health care providers add a rich context to define both challenges and opportunities for technology. To address user needs and expectations, technology must support routines that vary in style of intervention, level of independence, and ability to inspire habituation. The findings also showed that technology will provide the most value when it is able to adapt to unanticipated changes in these variables.

## Appendix

Multimedia Appendix 1 Survey questions used in study 1.

Multimedia Appendix 2 Semistructured interview questions for the medication taker interviews (study 2).

Multimedia Appendix 3 Semistructured interview questions for the health care provider interviews (study 3).

## Abbreviations

AI: artificial intelligence
FDA: Food and Drug Administration
HIPAA: Health Insurance Portability and Accountability Act of 1996
IoT: Internet of Things
ML: machine learning
PDR: Pre-Departure Reminder
PN: Proximity Notification
PN-Care: Proximity Notifications to Caregiver
PrEP: pre-exposure prophylaxis
RCS: Routine Change Detection
RCS-Team: Routine Change Detection Supported with Healthcare Providers
WHO: World Health Organization
