# Supplementary material for: Toward the Design of Sensing-Based Medication Adherence Aids That Support Individualized Activities of Daily Living: Survey and Interviews With Patients and Providers
Source: JMIR Hum Factors. 2023 Jul 4;10:e40173. doi: 10.2196/40173 (PMC10354653; doi:10.2196/40173)
Supplement: Multimedia Appendix 1 [file humanfactors_v10i1e40173_app1.pdf]

Appendix A – Survey questions used in study

Q57 Are you currently taking a medication that has been prescribed by a medical professional?

- ○ Yes
- ○ No

Display This Question:

If Are you currently taking a medication that has been prescribed by a medical professional? = No

Q58 Unfortunately you do not qualify for this study. Thanks for your interest.

Skip To: End of Survey If Unfortunately you do not qualify for this study. Thanks for your interest. Is Displayed

Q42 We will begin with questions which focus on practices and tools you use to organize your medications.

Q2 Do you use a pill box or pill organizer?

- ○ Always
- ○ Most of the time
- ○ About half the time
- ○ Sometimes
- ○ Never

Display This Question:

If Do you use a pill box or pill organizer? != Never

Q3 Where do you keep your pill organizer?

____

Display This Question:

If Do you use a pill box or pill organizer? != Never

Q4 Do you feel your pill organizer is effective in helping you manage our medications?

- ○ Extremely effective
- ○ Very effective
- ○ Moderately effective
- ○ Slightly effective
- ○ Not effective at all

Display This Question:

If Do you use a pill box or pill organizer? != Never

Q5 In your own words, please explain your effectiveness rating above.

____

____

____

____

____

Display This Question:

If Do you use a pill box or pill organizer? = Never

Q6 In your own words, please explain why you do not use a pill organizer.

____

Q7 Do you use a calendar or diary to track when you take your medications?

- Always
- Most of the time
- About half the time
- Sometimes
- Never

Display This Question:

If Do you use a calendar or diary to track when you take your medications? != Never

Q8 Do you feel your calendar or diary is effective in helping you manage your medications?

- Extremely effective
- Very effective
- Moderately effective
- Slightly effective
- Not effective at all

Display This Question:

If Do you use a calendar or diary to track when you take your medications? != Never

Q9 In your own words, please explain your effectiveness rating above.

____

____

____

____

____

Display This Question:

If Do you use a calendar or diary to track when you take your medications? = Never

Q10 In your own words, please explain why you do not use a calendar or diary.

____

____

____

____

____

Q11 Have you ever used, in the past or currently, any applications on your smart phone to mange your medications? For instance, a dedicated medication tracking application, specific medication tracking features of a broader health app, or apps provided by a health care provider or pharmacy.

○ Yes

○ No

Display This Question:

If Have you ever used, in the past or currently, any applications on your smart phone to mange your... = Yes

Q12 Which applications have you used (specific application names)?

____

Display This Question:

If Have you ever used, in the past or currently, any applications on your smart phone to mange your... = Yes

Q13 What features of the application or applications do or did you find useful?

Display This Question:

If Have you ever used, in the past or currently, any applications on your smart phone to mange your... = Yes

Q14 What features of the application or applications do or did you not find useful?

Display This Question:

If Have you ever used, in the past or currently, any applications on your smart phone to mange your... = No

Q15 For which reasons do you not use smart phone applications to manage your medications? (Select all that apply.)

- [ ] Don't have or want a smart phone
- [ ] Features I want do not exist
- [ ] Use of smart phone applications is too cumbersome
- [ ] Other people assist in managing medications and a smart phone application is difficult to share
- [ ] Smart phone is not used or is not near when taking medications
- [ ] Privacy and/or security concerns
- [ ] Something else

Display This Question:

If For which reasons do you not use smart phone applications to manage your medications? (Select all... = Features I want do not exist

Q16 What features do you believe are missing in current applications?

____

____

____

____

____

Display This Question:

If For which reasons do you not use smart phone applications to manage your medications? (Select all... = Other people assist in managing medications and a smart phone application is difficult to share

Q17 What other people assist in your medications? (Select all that apply.)

- ▢ Spouse
- ▢ Parent
- ▢ Adult child or children
- ▢ Adolescent child or children
- ▢ Friend
- ▢ Hired caregiver
- ▢ Medical professional (e.g. nurse or nursing assistant)
- ▢ Other

Display This Question:

If What other people assist in your medications? (Select all that apply.) = Other

Q18 Can you please describe your relationship with the person or persons who assist in your medications?

____

____

____

____

____

Display This Question:

If For which reasons do you not use smart phone applications to manage your medications? (Select all... = Something else

Q19 What other concerns do you have about using smart phone applications to manage your medications?

____

____

____

____

____

Q43 Next, we will ask about the situations and circumstances when medication doses are missed. Below we describe several situations and circumstances where adherence to taking a medication may be challenging. For each scenario please indicate how often these situations occur for you.

Q44 The medication was left in a different physical location. For example, the medicine was at home but needs to be taken when at work or when running errands.

- ◯ Never
- ◯ At least once a year
- ◯ At least once a month
- ◯ At least once a week
- ◯ At least once a day
- ◯ Not applicable (e.g. this situation would not apply to my medication regime)

Q45 The medication supply had been consumed before a prescription refill can be obtained.

- ◯ Never
- ◯ At least once a year
- ◯ At least once a month
- ◯ At least once a week
- ◯ At least once a day

Q46 Even though the medication was prescribed to be taken, it was not taken because symptoms were not present.

- ◯ Never
- ◯ At least once a year
- ◯ At least once a month
- ◯ At lease once a week
- ◯ At least once a day
- ◯ Not applicable (e.g. this situation would not apply to my medication regime)

Q51 Even though the medication was prescribed to be taken, it was not taken to avoid the drug's side effects.

- Never
- At least once a year
- At least once a month
- At least once a week
- At lease once a day
- Not applicable (e.g. this situation would not apply to my medication regime)

Q48 The dose was forgotten at the time medication needed to be taken.

- Never
- At least once a year
- At least once a month
- At least once a week
- At least once a day
- Not applicable (e.g. this situation would not apply to my medication regime)

Q51
Knew medication should be taken, but current activity prevents access or process for taking medication. For example, driving a car when a pill needs to be taken.

- Never
- At least once a year
- At least once a month
- At least once a week
- At least once a day
- Not applicable (e.g. this situation would not apply to my medication regime)

Q49 A social situation would make it embarrassing or impolite to take at time prescribed.

- Never
- At least once a year
- At least once a month
- At least once a week
- At least once a day
- Not applicable (e.g. this situation would not apply to my medication regime)

Q52 The dose of the medication was skipped to allow for the prescription to last longer, e.g. to save on the cost of the medication.

- Never
- At least once a year
- At least once a month
- At least once a week
- At least once a day
- Not applicable (e.g. this situation would not apply to my medication regime)

Display This Question:

If Have you ever used, in the past or currently, any applications on your smart phone to mange your... = Yes

Q53 The application I use to manage my medication(s) did not remind me, did not remind me correctly, or was not available to provide the reminder (e.g. misplaced phone).

- Never
- At least once a year
- At least once a month
- At least once a week
- At least once a day
- Not applicable (e.g. this situation would not apply to my medication regime)

Display This Question:

If Do you use a pill box or pill organizer? != Never

Q54 The pill box I use to manage my medication(s) was misplaced, incorrectly filled, or was not available at time medication needed to be taken (e.g. you were not at the location where you keep your pill box).

- Never
- At least once a year
- At least once a month
- At least once a week
- At least once a day
- Not applicable (e.g. this situation would not apply to my medication regime)

Display This Question:

If Do you use a calendar or diary to track when you take your medications? != Never

Q55 The diary I use to manage my medication(s) was misplaced, had incorrect entries, or was not available at time medication needed to be taken (e.g. you were not at the location where you keep your diary).

- Never
- At least once a year
- At least once a month
- At least once a week
- At least once a day
- Not applicable (e.g. this situation would not apply to my medication regime)

Q31 A goal of our research is to better understand the potential value for smart home technologies to assist in medication adherence. The following questions seek not to describe a particular technology, but the potential capabilities and use cases for these technologies. For each hypothetical technology, we ask you to rate the perceived utility of this technology to you.

Q32 A smart home technology that would detect when you are near your medications. Paired with a schedule of medication doses, the system would provide in-situ notifications. The system could provide these notifications on an audio on a smart speaker, on a wearable device (e.g. smart watch), or through a smart phone notification.

- ○ Very useful
- ○ Somewhat useful
- ○ Not useful

Q33 Do you believe this potential technology is more or less useful than your existing medication adherence routines?

- ○ Much better
- ○ Somewhat better
- ○ About the same
- ○ Somewhat worse
- ○ Much worse

Q34 A smart home technology that would detect when you are about to leave your home without taking your scheduled medications. The system could provide audio notifications on a smart speaker, on a wearable device (e.g. smart watch), or through a smart phone notification.

- ○ Very useful
- ○ Somewhat useful
- ○ Not useful

Q35 Do you believe this potential technology is more or less useful than your existing medication adherence routines?

- Much better
- Somewhat better
- About the same
- Somewhat worse
- Much worse

Q36 A smart home technology that would detect when a caregiver, family member, or person you trust to assist in your wellbeing is near your medications. The system could provide audio notifications on a smart speaker, on a wearable device (e.g. smart watch), or through a smart phone notification.

- Very useful
- Somewhat useful
- Not useful

Q37 Do you believe this potential technology is more or less useful than your existing medication adherence routines?

- Much better
- Somewhat better
- About the same
- Somewhat worse
- Much worse

Q38 A smart home technology that would learn more about your behaviors and movements when you are within and away from your home. It could use this information to suggest times and locations for taking medications that could lead to improved adherence.

- ◯ Very useful
- ◯ Somewhat useful
- ◯ Not useful

Q39 Do you believe this potential technology is more or less useful than your existing medication adherence routines?

- ◯ Much better
- ◯ Somewhat better
- ◯ About the same
- ◯ Somewhat worse
- ◯ Much worse

Q40 A smart home technology that would learn more about your behaviors and movements when you are within and away from your home. Behaviors and movements would be summarized and made available to healthcare professionals who provide you care. These summaries could be used to improve medication selection, scheduling, dosing, and other instructions by your healthcare professionals to improve adherence.

- ◯ Very useful
- ◯ Somewhat useful
- ◯ Not useful

Q41 Do you believe this potential technology is more or less useful than your existing medication adherence routines?

- Much better
- Somewhat better
- About the same
- Somewhat worse
- Much worse

Q56 A wearable or smart home technology that would learn more about your behaviors to classify when you are eating a meal. The technology could help remind you to take medications that need to be taken with a meal, or simply help you establish a routine of taking medications with meals.

- Very useful
- Somewhat useful
- Not useful

Q57 Do you believe this potential technology is more or less useful than your existing medication adherence routines?

- Much better
- Somewhat better
- About the same
- Somewhat worse
- Much worse

Q20 Thank you for providing information about your medication practices with technology. We would now like to gather details on the number, frequency, and schedule of the medications you take. We do not need specific medication details (e.g. names of the medicines or the doctors that prescribed them).

Q59 What is your age?

- ○ Under 18
- ○ 18 - 24
- ○ 25 - 34
- ○ 35 - 44
- ○ 45 - 54
- ○ 55 - 64
- ○ 65 - 74
- ○ 75 - 84
- ○ 85 or older

Q21 How many medications do your current take?

____

Display This Question:

If If How many medications do your current take? Text Response Is Greater Than 0

Q22 Using the table below, please indicate the number of medications you take at the times listed.

- 12AM - 6AM ____
- 6AM - 9AM ____
- 9AM-12PM ____
- 12PM-3PM ____
- 3PM-6PM ____
- 6PM - 9PM ____
- 9PM-12AM ____

Display This Question:

If If How many medications do your current take? Text Response Is Greater Than 0

Q23 Do you have any medications that must be taken separate from another drug?

- Yes
- No

Display This Question:

If If How many medications do your current take? Text Response Is Greater Than 0

Q24 Do you have any medications that must be taken with food or at meal time?

- Yes
- No

Q25 It is common to see multiple doctors as they specialize in different areas of medicine. How many doctors of different specialty have you seen in the past year?

- ○ 0
- ○ 1
- ○ 2
- ○ 3
- ○ 4
- ○ 5
- ○ 6+

Q26 How many of these doctors have prescribed a medicine or recommended an over the counter medicine, supplement, or vitamin.

- ○ 0
- ○ 1
- ○ 2
- ○ 3
- ○ 4
- ○ 5
- ○ 6+

Q27 Where do you keep your medications? (Select all that apply.)

- ☐ Bathroom
- ☐ Bedroom
- ☐ Kitchen
- ☐ On your person (carry with you)
- ☐ Outside the home
- ☐ Other

Display This Question:

If Where do you keep your medications? (Select all that apply.) = Outside the home

Q28 Where outside your home do you keep your medications?

____

____

____

____

____

Display This Question:

If Where do you keep your medications? (Select all that apply.) = Other

Q29 Can you please describe the other locations where you keep your medications?

____

____

____
