# Supplementary material for: Toward the Design of Sensing-Based Medication Adherence Aids That Support Individualized Activities of Daily Living: Survey and Interviews With Patients and Providers
Source: JMIR Hum Factors. 2023 Jul 4;10:e40173. doi: 10.2196/40173 (PMC10354653; doi:10.2196/40173)
Supplement: Multimedia Appendix 2 [file humanfactors_v10i1e40173_app2.pdf]

Appendix B - Semi-structured Interview Questions for Medication Taker Interviews

1. Describe your current health status.

This is to start the conversation and to help put the interviewees in a mental state to think about overall health and health practice. We are also trying to get a sense of the interviewees' overall perception of health as it relates to lifestyle and how dedicated they are to their current health goals.

[PROBES: How would you describe your overall health? Can you describe your current medical conditions? How is/are your medical condition(s) impacting your health? How are they impacting your lifestyle? Did your health change recently? Was the health change sudden or progressive?]

2. Describe which medications you take. Describe what each medication does for your health.

This question is about prescription literacy. We are trying to understand how active the interviewees are in managing their medications and how well they understand what the therapeutic benefits of the medication are.

[PROBES: Can you remember the names of all your medications without looking at the bottles? Which doctor prescribed those medicines? What is the specialty of that doctor? Where do you get your medicine? Why do you get it from there? Do you get your refills from the same place? Do you always go to the same location (e.g. always the same Walgreens)?]

3. Describe medications that are prescribed but not taken as prescribed.

This question is about obstacles that are beyond control of a potential technology intervention. Will help contextualize later comments on technology value.

[PROBES: Do you not believe the medication is important to your health? Is their concern about cost? Is it not possible to get to a pharmacy to fill the prescription?]

4. Describe when and where you take your medications.

We want to understand behaviors and practices around medication adherence, in particular the physical and contextual relationship of where prescription interactions occur within the broader home behaviors and activities.

[PROBES: Why do you keep your medications at that location? Did you used to keep your medications somewhere else? Why did you change locations? Did you think about putting them someplace else? Why do you feel this is a good location to keep your medications? Do you or have you considered keeping medications in more than one location? Does the location where you keep your medications change based upon the time of day or day of week? Why do you feel this time, and this place are the best for adhering to your medication?]

5. Describe how you use a pill organizer.

Pill organizers are an effective tool for managing and monitoring medication adherence. We want to understand past experiences with this tool in context of the health behaviors above as well as against the context of describing some potential technology interventions.

[PROBES: Why do you use a pill organizer? Who fills your pill organizer? If not you, why does that other person do this for you? Where and when is the pill organizer filled? Where do you keep your medication pill bottles in between filing your pill organizer? What are the benefits of the pill organizer? What about the pill organizer doesn't work well? Does using a pill organizer improve your adherence to taking your medication? How regularly do you use your pill organizer? Why do you sometimes stop using your pill organizer? Why do you choose not to use a pill organizer? If you don't use a pill organizer, have you used one in the past? Why did you stop using a pill organizer?]

5. Describe how you use a diary or calendar for the purpose of tracking your medications.

Like pill organizers, we are interested in existing organization and tracking behaviors.

[PROBES: Why do you use a diary or journal? Where do your journal or record your prescriptions? Is this a journal or calendar just for medications, or for other activities and events? Where do you keep this calendar or journal? Do you keep your journal or calendar in a digital tool? What about the journal or calendar works well? What doesn't work well? Does using a journal or calendar improve your adherence to taking your medication? Why don't you use a diary or journal? Have you ever considered using a diary or journal?]

6. Describe any existing technology tools used for the purpose of tracking your medications.

As above, interesting in existing organization and tracking behaviors. We are also interested in understanding current digital literacy around technology-based solutions.

[PROBES: What kind of phone do you use? Why did you pick this type of phone? What app do you most use on your phone? Do you use a pharmacy app on your phone? If yes, which ones? Do your refill prescriptions with this app? Do you also use the features of the pharmacy app to report your medications? Have you ever used an app solely dedicated to tracking medications? Do you use any apps for managing our tracking your health or exercise? Do you use other general-purpose apps (e.g., calendars, TODO lists, etc.) for tracking your medications?]

6b. Describe any wearable health devices use.

We are interested in understanding how literate the participants are in use if physical digital health tools.

[PROBES: What wearables do you use? Why do you use them? What functions of the device do you use? Do you feel these devices are effective for this use? How long have you been using this device? What doesn't work well? What feature do you believe is missing?]

6c. Describe any home devices or technologies used to monitor or maintain personal or environmental health.

We are interested in understanding how interested the participants are in the broader tech to support informed living and health.

[PROBE: Water filter, air purifier, air monitoring sensors, etc.]

7. Describe abstract concerns with using technology for tracking and managing health.

Before we describe specific technology interventions, we want to get a baseline understanding of interviewees' sentiments of using technology to support health activities and health practice.

[PROBES: How do you feel about technology being used to support health and wellness? Do you feel that the applications adequately protect your privacy? What assurances do you think technologies, and the organizations that supply and distribute them, should provide their users?]

Pause in interview – have interviewees show interviewers where medications are stored

* Interviewer will note and describe location. Ask interviewees to explain if location is shared with other members of the household. Ask to describe how reliably medication is kept in this location. Ask to describe typical activities that may take place at or around where medication is stored (if in a non-obvious location). Ask how often this location is visited in a given day by the interviewee; on weekdays, weekends.

*Interviewer will also work with the interviewee to sketch out a floor plan of their home. They will label the location where medications are stored, as well as places frequently used and for what purpose. For example, labeling a family room and describing activities such as watching TV, etc.

* Ask interviewees where other useful locations to keep medications in their home could be. Ask them to explain why they would be good locations. Ask them to compare to the location where they keep medication now. Ask to compare, contrast which would be better.

* Ask interviewees to walk the interviewer through morning and evening routines. This is both for routines where medications are being taken, or not taken.

We want to see location of where medications are stored to understand if information is consistent with responses to interview so far, as well as understand how much these locations are in agreement with responses received on surveys.

8. Describe who else lives in home.

We want to understand living situation as it relates to subsequent questions about other people assisting in medication adherence behaviors.

[PROBES: How long have you lived with this person or persons? If not a spouse, child, or parent, can you describe the relationship? Is it a trusting relationship? Does this person look out for your best interest?]

9. Describe other individuals that assist in medication adherence, organization, record keeping etc.

Our potential technologies touch on looping in other people close to the interviewees. We want to understand these relationships better and how they could influence the design of technologies that give them roles.

[PROBES: What does this person do to assist in your medications? Are they helpful with reminding you to take medication? Why do you suppose they are so helpful in remembering? Does this person help you organize your medications? Doe this person help with going to and navigating prescriptions and the pharmacy? Does this person go to the doctor with you when you get prescriptions? Does this person contact and communicate with doctors, nurses, or other health professionals on your behalf?]

10. Understand why cohabitators do not assist in medication adherence.

Survey results indicate that technologies that loop in others are less desired than other interventions. It is important to understand why this difference exists.

[PROBES: Why does this person(s) not share in the responsibilities? Do you not want to burden them? Do you want to maintain your privacy? Is this an activity that you believe should not be shared?]

Interview will now shift to describing potential technologies and asking for feedback.

*With each technology, we will ask interviewees to rate usefulness (very, somewhat, not).

* With each technology that is rated somewhat or very useful, we will ask interviewees to describe how that technology would work in their home. For instance, with technology for proximity, we will ask where that technology should be triggered for the given context.

*With each technology that is rated somewhat or very useful, we will ask interviewees how the technology could be further extended or enhanced to provide further value.

*With each technology that is rated not useful, we will ask the user to explain the reasons, and to also explain with the context of their preferences and living scenario.

* Interviewees will be probed to explain why a person may indicate an opposite result (e.g. useful when rating not useful). Interviewees will also be asked why they don't agree with that reasoning.

Technology scenarios:

"A smart home technology that would detect when you are near your medications. Paired with a schedule of medication doses, the system would provide in-situ notifications. The system could provide these notifications on an audio on a smart speaker, on a wearable device (e.g. smart watch), or through a smart phone notification."

"A smart home technology that would detect when you are about to leave your home without taking your scheduled medications. The system could provide audio notifications on a smart speaker, on a wearable device (e.g. smart watch), or through a smart phone notification."

"A smart home technology that would detect when a caregiver, family member, or person you trust to assist in your wellbeing is near your medications. The system could provide audio notifications on a smart speaker, on a wearable device (e.g. smart watch), or through a smart phone notification."

“A smart home technology that would learn more about your behaviors and movements when you are within and away from your home. It could use this information to suggest times and locations for taking medications that could lead to improved adherence.”

“A smart home technology that would learn more about your behaviors and movements when you are within and away from your home. Behaviors and movements would be summarized and made available to healthcare professionals who provide you care. These summaries could be used to improve medication selection, scheduling, dosing, and other instructions by your healthcare professionals to improve adherence.”

“A wearable or smart home technology that would learn more about your behaviors to classify when you are eating a meal. The technology could help remind you to take medications that need to be taken with a meal, or simply help you establish a routine of taking medications with meals.”
