# Supplementary material for: Toward the Design of Sensing-Based Medication Adherence Aids That Support Individualized Activities of Daily Living: Survey and Interviews With Patients and Providers
Source: JMIR Hum Factors. 2023 Jul 4;10:e40173. doi: 10.2196/40173 (PMC10354653; doi:10.2196/40173)
Supplement: Multimedia Appendix 3 [file humanfactors_v10i1e40173_app3.pdf]

Appendix C - Semi-structured Interview Questions for Healthcare Provider Interviews

1. Describe your level of concern regarding your patients' current hypertension medication adherence.

This is to start the conversation and help put the interviewees in a mental state to think about their patients' current hypertension medication adherence. We are also trying to gather information about the importance of a patient taking their hypertension medication as prescribed.

[PROBES: Literature suggests hypertension patients have a 50% medication adherence rate, is this reflected in your patients? How would you describe your patients' overall medication adherence? How important is medication adherence to a patient's overall health? How important is medication adherence to a patient's maintenance of hypertension? What are the health risks associated with non-adherence? Can you describe the connection between these health risks and non-adherence? Do your patients understand the importance of medication adherence in treating hypertension? Are there particular types of patients who do not understand the importance of medication adherence in treating hypertension?]

2. Describe the obstacles your patients face when maintaining hypertension medication adherence.

We want to understand the potential reasons why a patient is not taking their hypertension medication as prescribed.

[PROBES: What obstacles do your patients face in remaining adherent? Are there particular types of patients who are not adherent? Do these patients fall into specific socioeconomic categories? Do these patients fall into specific age groups? Do these patients have any other common traits? What are some indications that a patient is not taking their hypertension medication(s) as prescribed? How do you inquire if a patient is taking their medication(s) as prescribed? Do you ask the patients about their medication routines? What reason(s) do the patients tell you when they admit to not being adherent? Do patients place blame on themselves for not remaining adherent? In what ways do you build trust with your patients, so they are comfortable answering these questions?]

3. Describe your methods to promote medication adherence.

We want to understand what methods the physician has used and currently use to help their patients stay adherent.

[PROBES: What methods do you use to help your patients maintain medication adherence? What methods do you perceive to be successful in promoting adherence? Can you describe how these methods are successful? What types of patients benefit from these methods? What methods do you perceive to be unsuccessful in promoting adherence? Can you describe how these methods are unsuccessful? What types of patients do not benefit from these methods? Would your patients benefit from a technology that promotes medication adherence? What types of patients would find this technology beneficial? Can you describe how this technology would be beneficial for these patients? What types of patients would not find this technology beneficial? Can you describe how this technology would not be beneficial for these patients?]

Interview will now shift to describing potential technologies and asking for feedback.

*With each technology, we will ask interviewees to rate usefulness for their patients (very, somewhat, not).

* With each technology that is rated somewhat or very useful, we will ask interviewees to describe how that technology would work for their patients.

*With each technology that is rated somewhat or very useful, we will ask interviewees how the technology could be further extended or enhanced to provide added value to their patients.

*With each technology that is rated not useful, we will ask the interviewee to explain why it would not be useful for their patients.

* Interviewees will be probed to explain why a patient may indicate an opposite result (e.g., useful when rating not useful). Interviewees will also be asked why they disagree with that reasoning.

Technology scenarios:

“A smart home technology that would detect when a patient is near their medications. Paired with a schedule of medication doses, the system would provide in-situ notifications. The system could provide these notifications on an audio on a smart speaker, on a wearable device (e.g. smart watch), or through a smart phone notification.”

“A smart home technology that would detect when a patient is about to leave their home without taking your scheduled medications. The system could provide audio notifications on a smart speaker, on a wearable device (e.g. smart watch), or through a smart phone notification.”

“A smart home technology that would detect when a caregiver, family member, or person you trust to assist in your wellbeing is near your medications. The system could provide audio notifications on a smart speaker, on a wearable device (e.g. smart watch), or through a smart phone notification.”

“A smart home technology that would learn more about a patient behaviors and movements when they are within and away from their home. It could use this information to suggest times and locations for taking medications that could lead to improved adherence.”

“A smart home technology that would learn more about a patient’s behaviors and movements when they are within and away from their home. Behaviors and movements would be summarized and made available to, you, their healthcare professionals who provide them care. These summaries could be used to improve medication selection, scheduling, dosing, and other instructions by you, the healthcare professionals, to improve adherence.”

“A wearable or smart home technology that would learn more about a patient’s behaviors to classify when they are eating a meal. The technology could help remind them to take medications that need to be taken with a meal, or simply help them establish a routine of taking medications with meals.”
